# Remnant preservation and stump suturing in anterior cruciate ligament reconstruction reduce tibial tunnel widening without compromising tunnel placement: A computed tomography‐based randomised controlled trial

**DOI:** 10.1002/jeo2.70962

**Published:** 2026-10-28

**Authors:** Mahmoud El‐Desouky, Ahmed Samir Elkalyoby, Amr Samir Rashwan, Amr Ibrahim Gaballah, Begad Hesham Abdelrazek

**Affiliations:** ^1^ Department of Orthopaedic Surgery, Faculty of Medicine Cairo University Cairo Egypt

**Keywords:** ACL reconstruction, computed tomography, remnant preservation, tunnel placement accuracy, tunnel widening

## Abstract

**Purpose:**

Tunnel widening remains a concern following anterior cruciate ligament reconstruction because it may adversely affect graft incorporation and complicate revision surgery. Remnant preservation has been proposed as a strategy to reduce tunnel widening, but its effect on postoperative tunnel remodelling remains uncertain. This study aimed to determine whether remnant preservation with tibial stump suturing reduces tunnel widening without compromising tunnel placement accuracy.

**Methods:**

In this prospective randomised controlled trial, 179 patients undergoing primary anterior cruciate ligament (ACL) reconstruction were randomised to remnant‐preserving reconstruction with tibial stump suturing (*n* = 88) or conventional stump resection (*n* = 91). Standardised three‐dimensional computed tomography was performed at 2 weeks and 1 year postoperatively. Tibial and femoral tunnel widening were calculated as the percentage change in tunnel diameter between scans, and tunnel placement accuracy was assessed using the computed tomography‐based quadrant method. The incidence of clinically significant tunnel widening (>20%) was also compared.

**Results:**

Baseline tunnel diameters were comparable between groups. At 1‐year follow‐up, remnant‐preserving ACL reconstruction resulted in significantly less tibial tunnel widening than stump resection (14.58 ± 4.49% vs. 19.74 ± 4.36%, *p* < 0.001), with a lower incidence of clinically significant widening (>20%) (11.4% vs. 25.3%; relative risk, 0.45; *p* = 0.016). Femoral tunnel widening was similar between groups (18.23 ± 2.92% vs. 18.35 ± 2.59%, *p* = 0.766). No clinically relevant differences in tunnel placement were observed between groups.

**Conclusion:**

Remnant‐preserving ACL reconstruction with tibial stump suturing selectively reduces tibial tunnel widening at 12 months without compromising anatomic tunnel placement accuracy. These findings support the use of remnant preservation when preservation of tibial tunnel integrity is a surgical consideration.

**Level of Evidence:**

Level I.

**Trial Registration:**

ClinicalTrials.gov, NCT07236593; registration date: November 15, 2025 (retrospectively registered).

## Graphical abstract

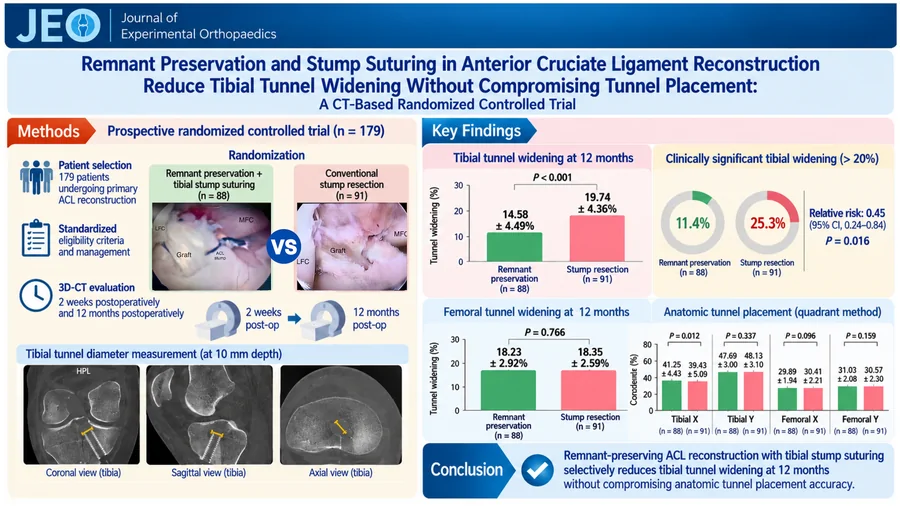

**Abbreviations**
3D‐CT: three‐dimensional computed tomography
ACL: anterior cruciate ligament
AP: anteroposterior
CSA: cross‐sectional area
CT: computed tomography
IKDC: International Knee Documentation Committee
MRI: magnetic resonance imaging
PCL: posterior cruciate ligament

## INTRODUCTION

Tunnel widening remains a recognised concern following anterior cruciate ligament (ACL) reconstruction because it may compromise graft fixation, interfere with tendon‐to‐bone healing, and increase the technical complexity of revision surgery [3, 16, 27]. Both mechanical and biological factors contribute to tunnel enlargement, including graft micromotion, synovial fluid ingress, stress shielding and inflammatory osteolysis. Recently, attention has shifted towards biological strategies aimed at enhancing tendon‐to‐bone healing and reducing tunnel expansion [9, 23, 30].

Preservation of the ACL remnant has been proposed as one such biological strategy because the retained tissue provides vascular synovium, mechanoreceptors, and a native scaffold that may facilitate graft healing [6, 8, 22, 28]. In addition, the preserved remnant may act as a biological seal that limits synovial fluid ingress into the tibial tunnel, potentially reducing tunnel remodelling [14, 15, 29]. Previous studies have reported reduced tunnel widening with remnant‐preserving techniques [14, 17, 29, 31]. However, whether this protective effect is more pronounced in the tibial tunnel remains unclear.

In the remnant‐preserving technique evaluated in the present study, the retained ACL stump was preserved at its tibial attachment and sutured to the reconstructed graft. Therefore, any local biological effect of remnant preservation would be expected to be more pronounced at the tibial tunnel than at the femoral tunnel [32]. Furthermore, although remnant preservation may provide potential biological advantages, the effect of retained remnant tissue on accurate anatomic tunnel placement remains an important technical consideration [13]. Therefore, whether remnant preservation with tibial stump suturing can reduce tibial tunnel widening while maintaining accurate anatomic tunnel placement warrants further investigation.

Therefore, this randomised controlled trial aimed to determine whether remnant‐preserving ACL reconstruction with tibial stump suturing selectively reduces tibial tunnel widening without compromising tunnel placement accuracy using standardised three‐dimensional computed tomography (3D‐CT). It was hypothesised that remnant preservation would selectively reduce tibial tunnel widening while maintaining accurate anatomic tunnel placement.

## METHODS

### Study design and included cases

This prospective randomised controlled study included 190 patients who underwent ACL reconstruction between July 2021 and March 2024 after approval by the institutional Research Ethics Committee. Patients with an ACL rupture undergoing primary ACL reconstruction were eligible for inclusion. Concomitant meniscal injuries were permitted. Exclusion criteria were skeletal immaturity, multiligamentous knee injury, previous ACL reconstruction, significant lower‐limb malalignment, and absence of an identifiable ACL stump suitable for preservation.

Based on pilot data, tibial tunnel widening was estimated to be 37.02% ± 27.60% in Group A and 48.71% ± 26.49% in Group B, corresponding to an anticipated between‐group difference of 11.69%. The required sample size was calculated using R software (version 4.3.1) and the pwr.t.test function from the pwr package. Assuming 80% power and a two‐sided significance level (*α* = 0.05), 86 participants were required per group. To compensate for an anticipated 10% loss to follow‐up, the target sample size was increased to 190 participants.

Participants were randomly assigned in a 1:1 ratio to either the remnant‐preservation group (Group A) or the stump‐resection group (Group B) using block randomisation. The allocation sequence was generated by an independent investigator who was not involved in patient recruitment, surgery, or outcome assessment. Allocation concealment was ensured using sequentially numbered, sealed opaque envelopes, which were opened only after patient enrollment. Group A underwent remnant‐preserving ACL reconstruction with tibial stump suturing, whereas Group B underwent conventional ACL reconstruction following complete stump resection.

After losses to follow‐up, 179 patients were available for the final analysis, including 88 patients in Group A and 91 patients in Group B (Figure 1). The study was conducted and reported in accordance with the Consolidated Standards of Reporting.

**Figure 1:**
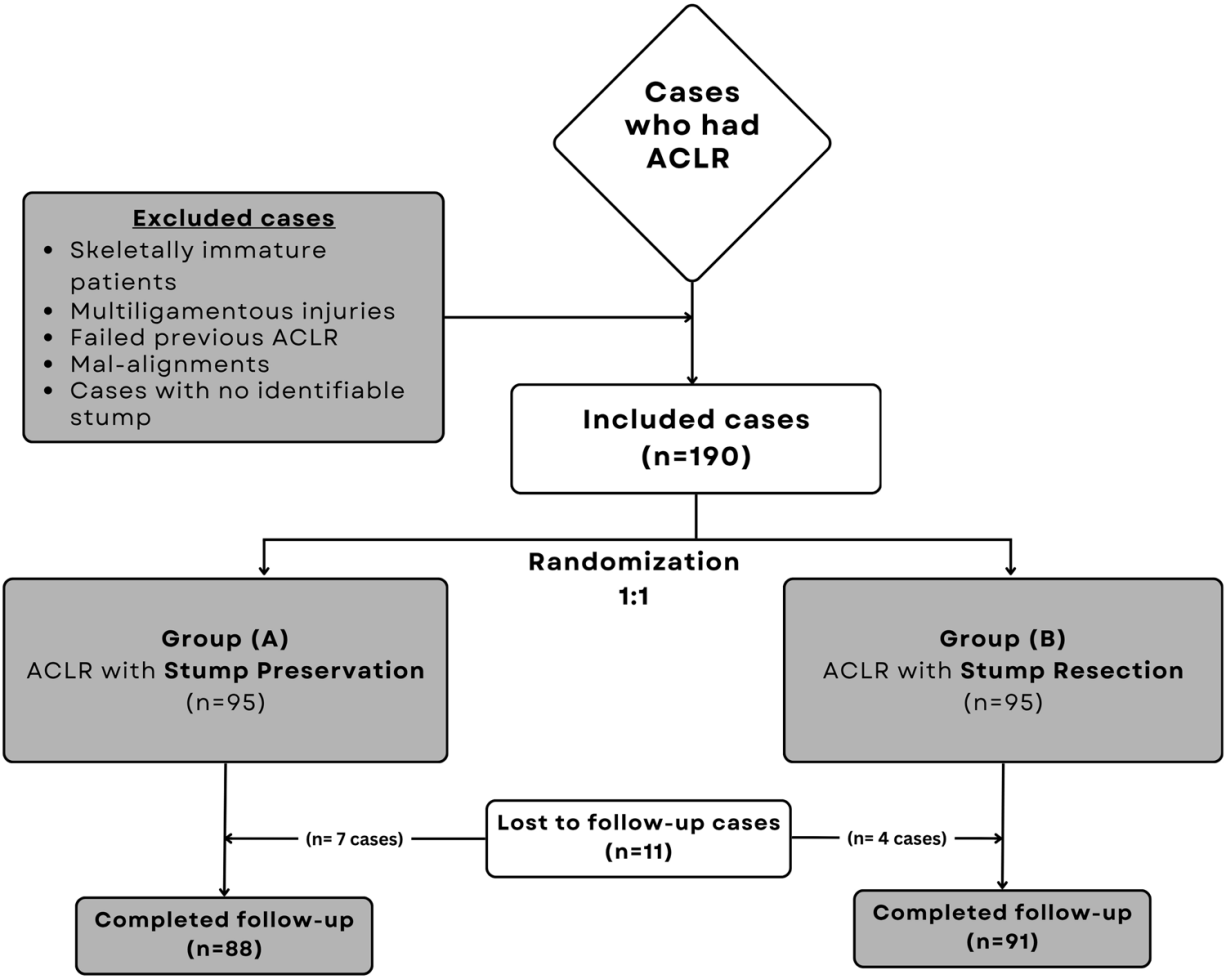
Flow chart of included cases. ACLR, anterior cruciate ligament reconstruction.

### Surgical technique

All procedures were performed using a standardised surgical technique by two consultant knee surgeons, each with more than 10 years of experience in ACL reconstruction before study initiation. Hamstring tendon autografts were used in all patients. Apart from remnant management, all surgical steps, fixation methods, and the postoperative rehabilitation protocol were identical between the two groups.

Anatomic femoral tunnel drilling was performed with the knee flexed to 120°. The femoral tunnel centre was positioned approximately 40% along the proximal‐to‐distal dimension of the lateral intercondylar notch and centred between the lateral intercondylar ridge and the posterior margin of the lateral femoral condyle. A guide pin was inserted, followed by reaming to match the diameter of the proximal part of the graft.

For tibial tunnel preparation, the tunnel was centred within the preserved tibial stump in the remnant‐preservation group. In the stump‐resection group, the tibial tunnel was targeted at the level of the posterior border of the anterior horn of the lateral meniscus, approximately 7 mm anterior to the posterior cruciate ligament. The tibial tunnel was reamed to match the graft diameter.

After graft passage, the preserved tibial stump in the remnant‐preservation group was tensioned using an arthroscopic grasper and approximated to the graft. A No. 1 Prolene suture was passed through both the ACL stump and the graft and tied arthroscopically, securing the preserved stump to the graft (Figure 2). Absence of graft impingement was confirmed arthroscopically with the knee in extension. Femoral fixation was achieved using suspensory cortical fixation (ToggleLoc with ZipLoop; Zimmer Biomet, Warsaw, IN, USA, or ACL TightRope II; Arthrex, Naples, FL, USA), whereas tibial fixation was performed using bioabsorbable interference screws (Mitek Interference Screw; DePuy Synthes, Raynham, MA, USA) 1 mm larger than the graft diameter.

**Figure 2:**
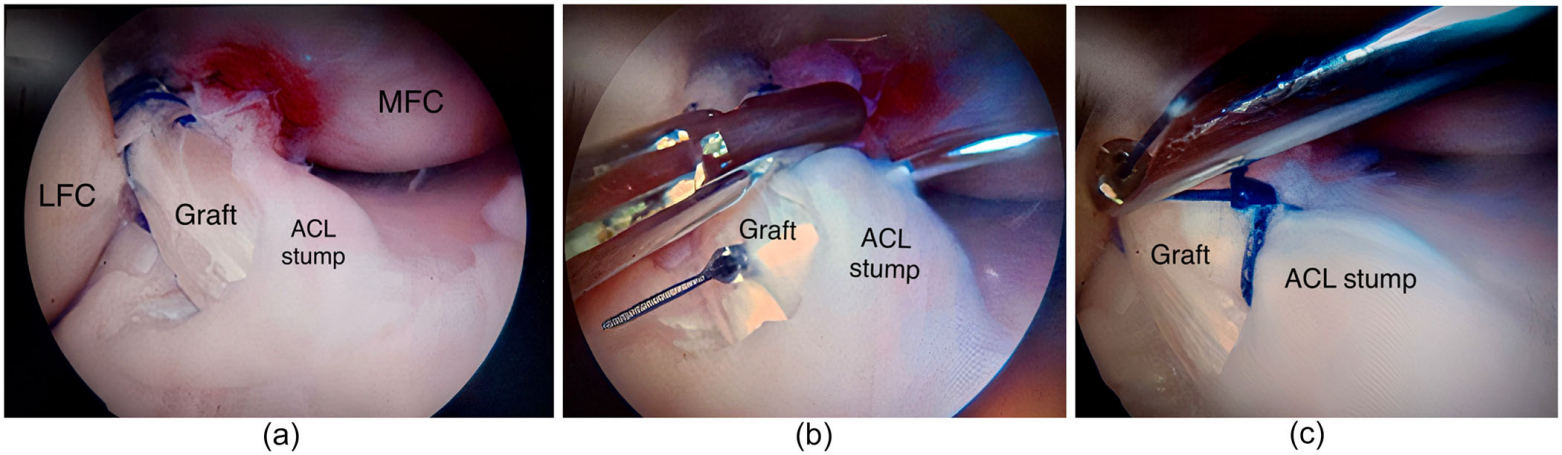
Arthroscopic steps of the remnant‐preservation and stump‐suturing technique. (a) Graft passage through the preserved tibial stump. (b) Passage of a No. 1 Prolene suture through the ACL stump and graft. (c) Final appearance after arthroscopic tying of the stump to the graft. Reproduced from Reference [7] under the Creative Commons Attribution 4.0 International License. ACL, anterior cruciate ligament; LFC, lateral femoral condyle; MFC, medial femoral condyle.

### Postoperative rehabilitation and follow‐up

All patients followed the same standardised postoperative rehabilitation protocol, irrespective of the allocated treatment group. During the first 2 postoperative weeks, rehabilitation focused on pain and swelling control, restoration of full knee extension, progressive recovery of knee flexion, quadriceps activation, patellar mobilisation and straight‐leg raising. Weight bearing was allowed as tolerated with crutches and gradually progressed to full weight bearing according to patient tolerance.

From 2 to 6 weeks, progressive range of motion exercises were continued with the aim of restoring full knee motion, together with progressive closed‐chain quadriceps strengthening. Thereafter, strengthening exercises were progressively advanced according to clinical recovery.

In patients who underwent concomitant meniscal repair, the rehabilitation protocol was modified to protect the repair. Knee flexion was restricted to 90° and protected partial weight bearing with crutches was maintained during the first 4–6 postoperative weeks. Thereafter, gradual progression to full weight bearing and unrestricted range of motion was allowed.

Patients underwent regular clinical follow‐up every 2 weeks during the first 3 postoperative months and monthly thereafter until completion of follow‐up.

### Outcomes

The primary outcome was tibial tunnel widening at 1 year, expressed as the percentage increase in tunnel diameter relative to the early postoperative CT scan.

Secondary outcome included femoral tunnel widening, the incidence of clinically significant tunnel widening (>20%) in both tunnels, and tunnel placement accuracy assessed using the CT‐based quadrant method.

### CT imaging protocol

All patients underwent non‐contrast CT examinations at 2 weeks postoperatively to establish baseline femoral and tibial tunnel diameters and tunnel positions. CT imaging was repeated at 1 year to evaluate tunnel widening.

CT imaging was performed using a 64‐slice multidetector scanner (Somatom Definition AS+, Siemens Healthineers, Germany). Acquisition parameters were: collimation 64 × 0.6 mm, slice thickness 0.6 mm, 120 kV, 100 mA, matrix 512 × 512, and field of view 170 mm. Axial datasets were reconstructed into multiplanar images (oblique axial, oblique sagittal, and oblique coronal planes) and converted into 3D volume‐rendered images using Syngo.via workstation.

All CT measurements were independently performed by two blinded observers in separate sessions, and the average of both independent measurements was used for the final analysis.

#### Measurement of tunnel widening

Tunnel widening was quantified by comparing tunnel diameters measured on the early postoperative (2‐week) and 1‐year CT scans. Measurements were obtained 10 mm from the intra‐articular tunnel aperture, where postoperative tunnel remodelling is most consistently observed while minimising the influence of aperture irregularities and fixation devices.

For each tunnel, diameters were measured on oblique axial, oblique sagittal, and oblique coronal multiplanar reconstructions perpendicular to the tunnel axis (Figure 3). The mean of the three measurements was considered the tunnel diameter at each time point.

**Figure 3:**
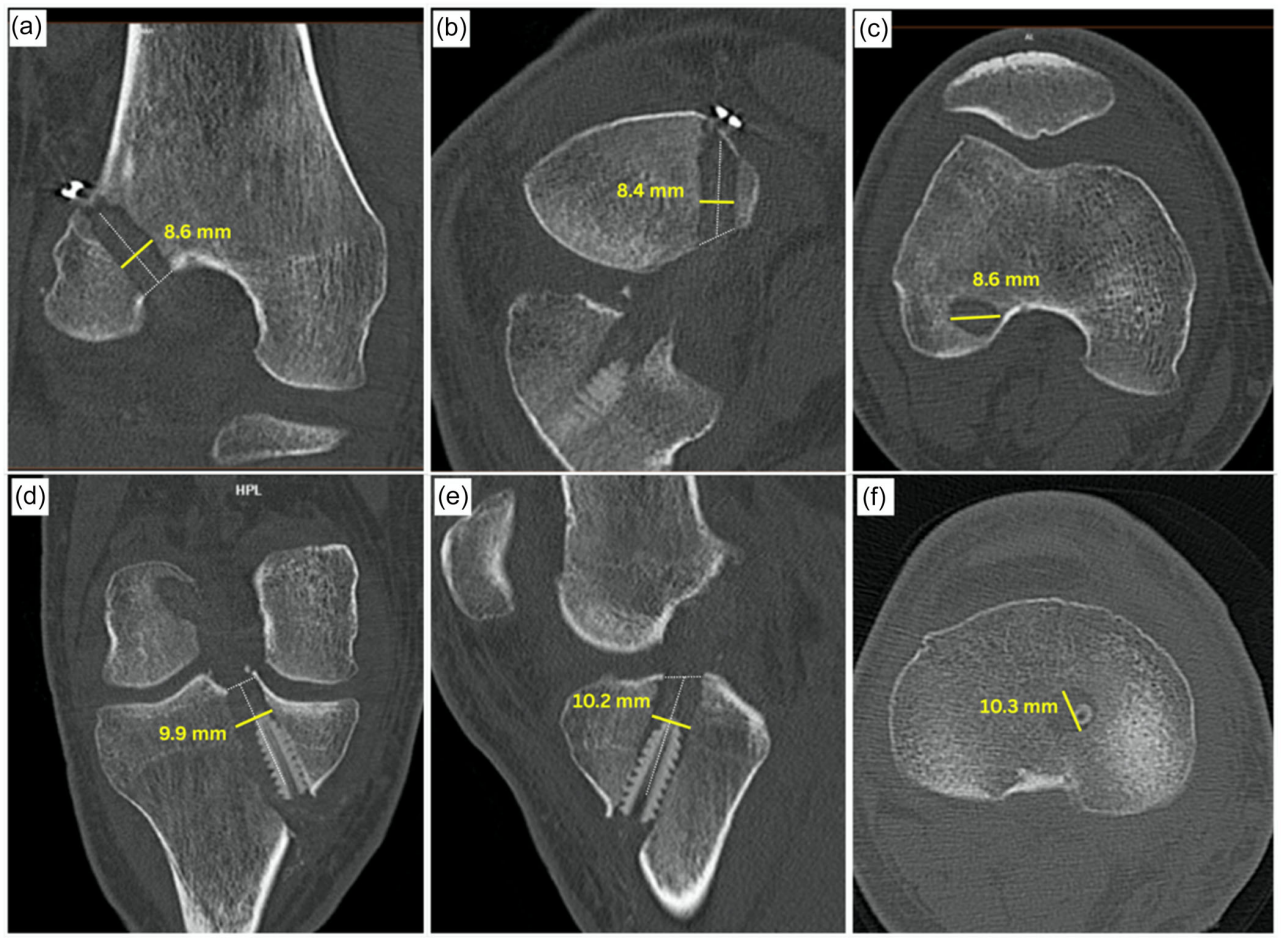
Tunnel diameter measured 10 mm from the intra‐articular aperture with lines drawn perpendicular to the tunnel axis. (a–c) Femoral tunnel diameter calculated as the average of three orthogonal measurements (coronal, sagittal, axial). (d–f) Tibial tunnel diameter calculated similarly as the average of three orthogonal measurements.

Tunnel widening was calculated as the percentage increase in tunnel diameter at 1 year relative to the early postoperative CT measurement. A tunnel widening of >20% was predefined as clinically significant based on previous literature [10, 17, 18].

### Assessment of tunnel position

Femoral and tibial tunnel positions were measured using the quadrant method on the 3D CT scan images obtained 2 weeks postoperatively [4, 25].

For the tibial tunnel, measurements were performed on the en‐face view of the tibial plateau. The *x*‐axis was defined along the anteroposterior dimension of the tibial plateau, with its origin at the anterior edge and positive direction towards the posterior edge. The *x*‐coordinate of the tunnel centre was calculated as the distance from the anterior edge to the projected tunnel centre divided by the total anteroposterior length of the plateau and expressed as a percentage. The *y*‐axis was defined along the mediolateral dimension of the tibial plateau, with its origin at the medial edge and positive direction towards the lateral edge. The *y*‐coordinate was calculated as the distance from the medial edge to the projected tunnel centre divided by the total mediolateral width of the plateau and expressed as a percentage (Figure 4) [25].

**Figure 4:**
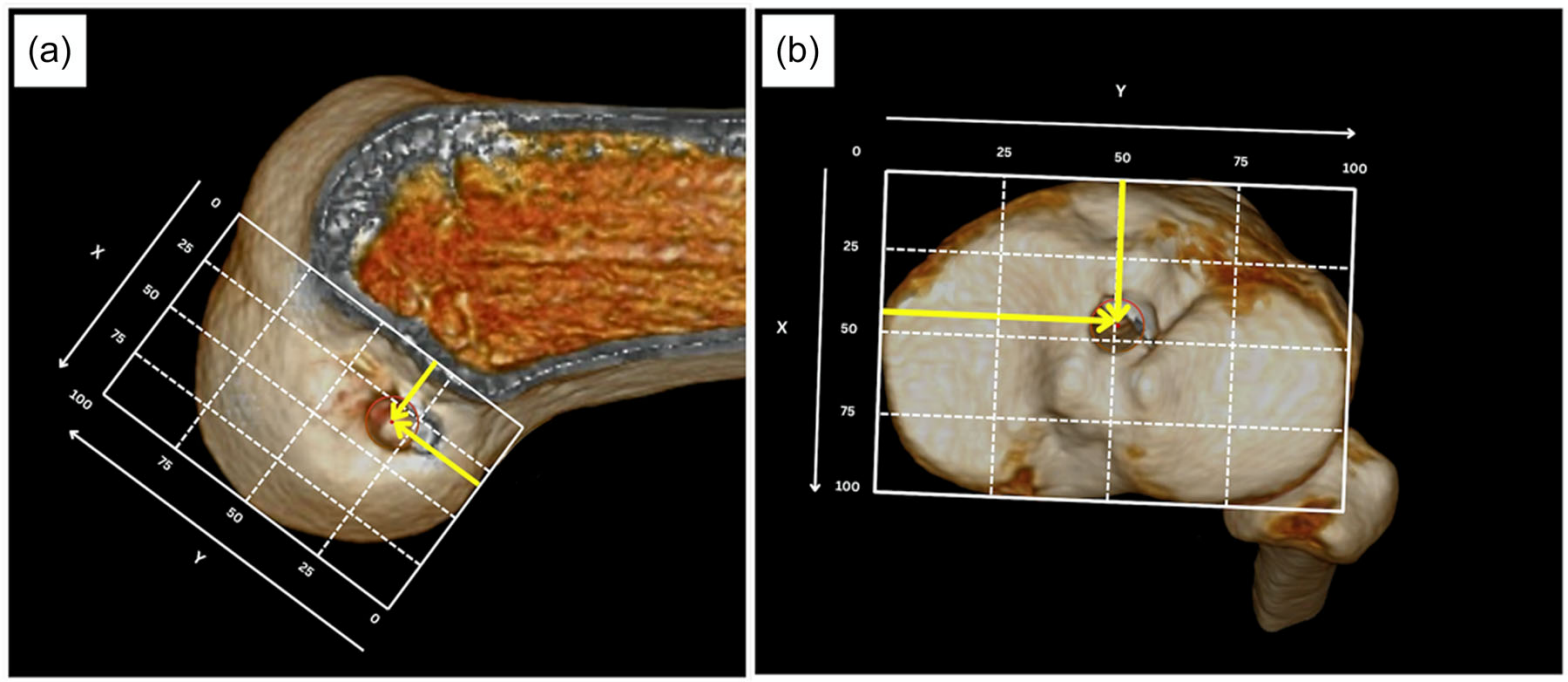
Three‐dimensional computed tomography assessment of tunnel position using the quadrant method at 2 weeks postoperatively. (a) Femoral tunnel position: the *X* axis extends from superior to inferior, with its origin at the superior edge of the lateral femoral condyle, whereas the *Y* axis extends from deep to shallow, with its origin at the posterior (deep) edge. The *x*‐ and *y*‐coordinates of the tunnel centre are expressed as percentage distances along the total supero‐inferior height and anteroposterior depth of the lateral femoral condyle, respectively. (b) Tibial tunnel position: the *X* axis extends from anterior to posterior, with its origin at the anterior edge of the tibial plateau, whereas the *Y* axis extends from medial to lateral, with its origin at the medial edge. The *x*‐ and *y*‐coordinates of the tunnel centre are expressed as percentage distances along the total anteroposterior length and mediolateral width of the tibial plateau, respectively.

For the femoral tunnel, measurements were performed on the intra‐articular surface of the lateral femoral condyle. The *x*‐axis was defined along the superior–inferior dimension of the lateral femoral condyle, with its origin at the superior edge and positive direction towards the inferior edge. The *x*‐coordinate of the tunnel centre was calculated as the distance from the superior edge to the projected tunnel centre divided by the total supero‐inferior height of the condyle and expressed as a percentage. The *y*‐axis was defined along the deep–shallow dimension, with its origin at the posterior (deep) edge and positive direction towards the anterior (shallow) edge. The *y*‐coordinate was calculated as the distance from the posterior edge to the projected tunnel centre divided by the total anteroposterior depth of the condyle and expressed as a percentage (Figure 4) [4].

### Measurement reliability

All tunnel diameter and tunnel position measurements were independently performed by two blinded observers in separate sessions. One observer repeated all measurements after a 4‐week interval to assess intraobserver reliability. For each parameter, the average of the two observers’ measurements was considered the final recorded value. Interobserver and intraobserver reliability were assessed using the intraclass correlation coefficient (ICC).

### Statistical analysis

Statistical analyses were performed using IBM SPSS Statistics for Windows, version 28.0 (IBM Corp.). Continuous variables are expressed as mean ± standard deviation (SD) and compared using the independent‐samples *t* test. Interobserver and intraobserver reliability were assessed using the intraclass correlation coefficient (ICC) with 95% confidence intervals, based on a two‐way random‐effects model with absolute agreement. ICC values > 0.75 were considered good, and >0.90 excellent. A two‐sided *p*‐value < 0.05 was considered statistically significant.

## RESULTS

A total of 179 patients were included in the final analysis, including 88 patients in the remnant‐preservation group and 91 patients in the stump‐resection group. Baseline demographic and injury characteristics were comparable between the two groups (Table 1).

**Table 1:** Baseline characteristics of included patients.

	Group A (Stump preservation) (*n* = 88)	Group B (Stump resection) (*n* = 91)
Age (years)	29.68 ± 5.63	28.70 ± 6.27
Gender		
Males	81 (92.0%)	86 (94.5%)
Females	7 (8.0%)	5 (5.5%)
Side		
Right	49 (55.7%)	61 (67.0%)
Left	39 (44.3%)	30 (33.0%)
Time to surgery (weeks)	13.23 ± 5.76	14.12 ± 6.85
Body mass index (kg/m^2^)	24.20 ± 2.83	23.92 ± 1.97
Concomitant meniscal injury		
Medial	28 (31.8%)	17 (18.7%)
Lateral	11 (12.5%)	16 (17.6%)
None	49 (55.7%)	58 (63.7%)
Meniscal procedure		
Repair	16/39 (41.0%)	12/33 (36.4%)
Partial meniscectomy	23/39 (59.0%)	21/33 (63.6%)

### Reliability analysis

Interobserver and intraobserver reliability were good to excellent for all tunnel diameter and tunnel position measurements. Interobserver intraclass correlation coefficients ranged from 0.79 to 0.92, whereas intraobserver coefficients ranged from 0.87 to 0.93.

### Tunnel Widening

Baseline tibial and femoral tunnel diameters measured 2 weeks postoperatively were comparable between the groups (Table 2). At 1‐year follow‐up, the remnant‐preservation group demonstrated significantly less tibial tunnel widening than the stump‐resection group (14.58 ± 4.49% vs. 19.74 ± 4.36%, *p* < 0.001), with a lower incidence of clinically significant (>20%) tibial tunnel widening (11.4% vs. 25.3%; relative risk, 0.45; *p* = 0.016). Femoral tunnel widening was comparable between the groups (18.23 ± 2.92% vs. 18.35 ± 2.59%, *p* = 0.766), with no significant between‐group difference in the incidence of clinically significant femoral tunnel widening (Table 2).

**Table 2:** Tibial and femoral tunnel widening measurements.

	Group A (Stump preservation) (*n* = 88)	Group B (Stump resection) (*n* = 91)	Absolute difference (Group B – Group A)	*p*‐value
Tibial tunnel diameter				
Tunnel diameter at 2 weeks (mm)	9.47 ± 0.60	9.56 ± 0.48	0.09 mm	0.270
Tunnel diameter at 1 year (mm)	10.84 ± 0.63	11.45 ± 0.73	0.61 mm	<0.001
Tunnel widening (%)	14.58 ± 4.49	19.74 ± 4.36	5.16%	<0.001
Incidence of tunnel widening (%)	10 (11.4%)	23 (25.3%)	13.90%	0.016
Femoral tunnel diameter				
Tunnel diameter at 2 weeks (mm)	8.42 ± 0.41	8.45 ± 0.50	0.03 mm	0.659
Tunnel diameter at 1 year (mm)	9.95 ± 0.42	10.00 ± 0.65	0.05 mm	0.514
Tunnel widening (%)	18.23 ± 2.92	18.35 ± 2.59	0.12%	0.766
Incidence of tunnel widening (%)	14 (15.9%)	16 (17.6%)	1.70%	0.764

### Tunnel position

Tunnel position measurements were largely comparable between the groups (Table 3). A statistically significant difference was observed only for the tibial *x*‐coordinate (41.25 ± 4.43% vs. 39.43 ± 5.09%, *p* = 0.012), whereas no significant differences were found for the remaining tibial or femoral tunnel coordinates.

**Table 3:** Tibial and femoral tunnel position.

	Group A (Stump preservation) (*n* = 88)	Group B (Stump resection) (*n* = 91)	Absolute difference (Group B – Group A) (%)	*p*‐value
Tibial tunnel position				
*X*‐Coordinate	41.25 ± 4.43	39.43 ± 5.09	1.82	0.012
*Y*‐Coordinate	47.69 ± 3.00	48.13 ± 3.10	0.44	0.337
Femoral tunnel position				
*X*‐Coordinate	29.89 ± 1.94	30.41 ± 2.21	0.52	0.096
*Y*‐Coordinate	31.03 ± 2.08	30.57 ± 2.30	0.46	0.159

## DISCUSSION

The present study represents a radiological extension of a previously published randomised clinical trial evaluating ACL remnant preservation [7]. While the original study focused on functional outcomes, proprioception, and postoperative complications, the current analysis used standardised three‐dimensional CT to investigate tunnel remodelling and tunnel placement accuracy.

The principal finding was that stump suturing selectively reduced tibial tunnel widening without affecting femoral tunnel widening or tunnel placement accuracy. Compared with stump resection, the remnant‐preservation group demonstrated significantly less tibial tunnel widening (14.6% vs. 19.7%) and a lower incidence of clinically significant tunnel widening (>20%) (11.4% vs. 25.3%). These findings suggest that preserving the native ACL stump may promote graft–bone healing by limiting synovial fluid ingress into the tibial tunnel.

Femoral tunnel widening was similar between the groups (18.2% vs. 18.3%), suggesting that any biological effect of remnant preservation is more pronounced on the tibial side. Greater tunnel–synovial communication has been proposed to contribute to tunnel enlargement potentially because gravity facilitates the flow of synovial fluid into the tibial tunnel and increases exposure of the graft–bone interface to synovial fluid and its inflammatory mediators [19, 20]. Additionally, the relatively lower bone density of the proximal tibia may render the tibial tunnel more susceptible to bone remodelling and subsequent tunnel enlargement [12, 24].

Tunnel position was also comparable between the groups. Although a statistically significant difference was observed for the tibial *x*‐coordinate (41.25% vs. 39.43%, *p* = 0.012), no significant differences were found for the remaining tibial or femoral coordinates, indicating that remnant preservation did not compromise anatomic tunnel placement.

Overall, remnant preservation with stump suturing reduced tibial tunnel widening while maintaining accurate tunnel placement.

The findings of the present study are broadly consistent with previous studies suggesting a protective effect of remnant preservation on tunnel biology. Zhang et al. reported that remnant preservation in single‐bundle ACL reconstruction reduced the mean percentage increase in tibial tunnel diameter from 34.0% to 25.7% and decreased the incidence of clinically significant tunnel widening from 58.3% to 29.6% [31]. Consistent with the findings of the present study, a protective effect of remnant preservation on the tibial tunnel was demonstrated.

However, their tunnel measurements were based on plain radiographs obtained up to 24 weeks postoperatively, whereas the present study used standardised three‐dimensional CT immediately after surgery and at 1 year. Given the higher accuracy of CT for evaluating bone tunnels and the continued remodelling reported up to 10 months postoperatively, the methodology used in the present study may provide a more reliable assessment of tunnel widening [5, 26].

Similar findings have also been reported in double‐bundle ACL reconstruction. Yanagisawa et al. and Masuda et al. demonstrated significantly less tunnel enlargement in remnant‐preserving reconstructions, particularly in the anteromedial femoral and tibial tunnels [17, 29]. Yanagisawa et al. also used serial CT measurements, making their methodology comparable to the present study. However, tunnel remodelling was assessed only up to 6 months, and they found that reduced tunnel widening was not associated with superior functional outcomes, an association that was beyond the scope of the current radiological analysis.

Koyama et al. further demonstrated reduced tunnel area expansion in all four tunnels after anatomic double‐bundle ACL reconstruction using a refined remnant‐preserving technique [14]. However, tunnel measurements in that study were obtained merely 2 mm from the tunnel aperture, whereas the present study evaluated tunnel diameter at a standardised depth of 10 mm from the aperture. Measurement at a depth of 10 mm provides a more accurate and reproducible assessment of true tunnel widening by avoiding superficial aperture‐related artifacts, irregular funneling and localised edge wear associated with tunnel drilling and graft micromotion, which may become more pronounced closer to the tunnel aperture [1, 19]. Similarly, Tachibana et al. recommended measurements 5 mm from the tunnel aperture but evaluated tunnel widening using axial CT images alone. In contrast, tunnel diameters in the present study were measured on axial, sagittal and coronal CT images, providing a more comprehensive assessment of tunnel morphology [21].

In contrast, Naraoka et al. did not observe a reduction in tibial tunnel enlargement with remnant preservation and even reported greater posterolateral (PL) axial widening in the remnant‐preserving group. They performed double bundle reconstructions and kept only the tibial stump and assessed tunnel position and diameter from CT and magnetic resonance imaging (MRI), which might show greater accuracy. However, tunnels in DB reconstructions are smaller and the original narrower tunnel diameter may affect tunnel widening. The posterolateral tunnel in the tibia is furthest away from the stump and this may explain their findings of greater widening in the PL tunnel [18].

Similarly, Kim et al. reported no significant increase in tunnel cross‐sectional area despite remnant preservation [11]. Although the mean tunnel area increased by 3.49 mm^2^, the overall change was not statistically significant, and many patients demonstrated a reduction in tunnel area. Their exclusive use of Achilles tendon allografts with bone plugs may have influenced graft–bone healing and limited tunnel enlargement. Consistent with this, Ashy et al. reported lower rates of tunnel widening with grafts incorporating bone blocks, including bone–patellar tendon–bone grafts [2].

Li et al. reported that preservation of the femoral remnant reduced femoral tunnel enlargement from 22.4% to 15.1% [15], in contrast to the present study, in which femoral tunnel widening was comparable between the groups. These differing findings may reflect important methodological differences, including preservation of the femoral rather than the tibial remnant, MRI‐based measurements at 6 months, and comparison with the intraoperative drill diameter rather than immediate postoperative tunnel measurements. Together, these observations suggest that the location of remnant preservation, imaging modality, and follow‐up duration may influence the apparent effect of remnant preservation on tunnel remodelling.

Regarding the tunnel position and accuracy and precision of tunnel placement, a study by Kosy et al. found no significant difference in tunnel positioning, accuracy and precision between both remnant preserving and sacrificing techniques. This is consistent with the findings of this current study and highlights that with good surgeon's experience, remnant preservation ACLR is reproducible [13].

The present findings therefore support the growing body of evidence indicating a beneficial effect of remnant preservation on tunnel widening and may help explain the heterogeneous results reported in the literature by suggesting that this protective effect is predominantly confined to the tibial tunnel.

The strengths of this study include its prospective randomised controlled design with allocation concealment, standardised surgical technique and rehabilitation protocol, and the use of high‐resolution CT at two predefined time points (2 weeks and 1 year). Tunnel diameter and position were independently assessed by two blinded reviewers, improving measurement reliability. Furthermore, the evaluation of both percentage tunnel enlargement and clinically significant widening (>20%) enhances the clinical relevance of the findings.

Several limitations should be acknowledged. This was a single‐centre study, which may limit the generalisability of the findings. Although a 1‐year follow‐up is appropriate for evaluating early tunnel remodelling, it does not capture long‐term tunnel behaviour or graft incorporation. Additionally, the intervention combined remnant preservation with stump suturing; therefore, the observed reduction in tibial tunnel widening cannot be attributed specifically to remnant preservation, stump suturing, or their combined effect. Future comparative studies evaluating remnant preservation with versus without stump suturing are warranted to clarify the independent contribution of stump suturing to tunnel remodelling. Finally, this radiological study did not examine the relationship between tunnel widening and clinical outcomes.

## CONCLUSION

Remnant‐preserving ACL reconstruction with stump suturing selectively reduced postoperative tibial tunnel widening at 12 months without affecting femoral tunnel widening or anatomic tunnel placement accuracy. This technique may therefore represent a useful surgical option for selectively limiting tibial tunnel remodelling during the first postoperative year while preserving accurate anatomic tunnel placement.

## AUTHOR CONTRIBUTIONS

All authors contributed to the study conception and design. Ahmed Samir Elkalyoby, Begad Hesham Abdelrazek and Amr Samir Rashwan performed the surgical procedures. Mahmoud El‐Desouky and Amr Ibrahim Gaballah collected and analysed the data. Mahmoud El‐Desouky drafted the manuscript. All authors critically revised the manuscript, read and approved the final version.

## FUNDING INFORMATION

The authors have no funding to report.

## CONFLICT OF INTEREST STATEMENT

The authors declare no conflicts of interest.

## ETHICS STATEMENT

The study was approved by the Research Ethics Committee, Faculty of Medicine, Cairo University (Approval No. N‐473‐2021). Written informed consent was obtained from all participants before inclusion in the study.

## Supporting information

CONSORT‐2010‐Checklist.

## Data Availability

The datasets generated and analysed during the current study are available from the corresponding author on reasonable request.
